# Early Investigational and Experimental Therapeutics for the Treatment of Hypertriglyceridemia

**DOI:** 10.3390/jcdd9020042

**Published:** 2022-01-25

**Authors:** Ioannis Parthymos, Michael S. Kostapanos, George Liamis, Matilda Florentin

**Affiliations:** 1 Department of Internal Medicine, School of Medicine, University of Ioannina, 45110 Ioannina, Greece; 5472ioannis@gmail.com (I.P.); gliamis@uoi.gr (G.L.); 2 Lipid Clinic, Department of General Medicine, Addenbrooke’s Hospital, Cambridge University Hospitals NHS Foundation Trust, Cambridge CB2 0QQ, UK; mixkos@hotmail.com

**Keywords:** triglycerides, apolipoprotein C-III, lipoprotein lipase, ω-3 fatty acids, volanesorsen, fibroblast growth 21 factor

## Abstract

Hypertriglyceridemia has been identified as a risk factor for cardiovascular disease and acute pancreatitis. To date, there are only few drug classes targeting triglyceride levels such as fibrates and ω-3 fatty acids. These agents are at times insufficient to address very high triglycerides and the residual cardiovascular risk in patients with mixed dyslipidemia. To address this unmet clinical need, novel triglyceride-lowering agents have been in different phases of early clinical development. In this review, the latest and experimental therapies for the management of hypertriglyceridemia are presented. Specifically, ongoing trials evaluating novel apolipoprotein C-III inhibitors, ω-3 fatty acids, as well as fibroblast growth 21 analogues are discussed.

## 1. Introduction

Hypertriglyceridemia, defined as triglycerides (TG) > 150 mg/dL (1.7 mmol/L), may be primary due to genetic disorders, or secondary in the context of other diseases, such diabetes mellitus (DM), alcoholism, chronic kidney disease, and endocrine disorders [1]. The risk for acute pancreatitis increases progressively with serum TG levels over 500 mg/dL (5.6 mmol/L) and more markedly with levels over 1000 mg/dL (11.3 mmol/L), whilst lower TG levels are encountered in atherogenic dyslipidemia and are associated with enhanced cardiovascular (CV) risk [2,3]. Microvascular complications, mostly renal disorders, are also more frequently encountered in individuals with elevated TG levels [4]. Namely, hypertriglyceridemia was identified as a significant residual CV risk contributor in statin-treated patients with acute coronary syndromes as well as in patients with DM [5,6,7,8]. Importantly, increased TGs may also be associated with premature subclinical atherosclerosis and vascular inflammation in apparently healthy individuals. This was shown in a recent trial which assessed peripheral atherosclerotic plaques with the use of two-dimensional vascular ultrasound and vascular inflammation with the use of positron emission tomography [9].

Several familial conditions are associated with markedly increased circulating TG levels and chylomicronemia (TG > 1000 mg/dL; 11.3 mmol/L), subsequently increasing the risk of acute pancreatitis [10]. Among these are rare monogenic disorders which severely impair lipoprotein lipase (LPL) activity, mutations leading to apolipoprotein (apo) C-II deficiency, as well as loss-of-function mutations in *APOA5* and *GPIHBP1*.

Conversely, some genetic disorders are associated with elevated TG levels and increased CV disease (CVD) risk; these include familial combined hyperlipidemia (FCHL) and type III dysbetalipoproteinemia. In contrast, familial hypertriglyceridemia (FHTG) has been less associated with increased CVD risk [11].

Most available lipid-lowering drugs have only modest TG-lowering effects, and the TG reduction they bring about has not been associated with clinically relevant reductions in CV outcomes [12]. Fibrates, the first-line agents for the treatment of severe hypertriglyceridemia, have not been consistently associated with significant reductions in CVD risk. Similar are the findings from studies evaluating existing omega-3 fatty acid formulations of eicosapentaenoic (EPA) and docosahexaenoic (DHA) [13]. In contrast, icosapent ethyl in the REDUCE-IT (cardiovascular risk reduction with icosapent ethyl for hypertriglyceridemia) study demonstrated favorable CV outcomes, as will be discussed later on. Besides, patients with monogenic disorders associated with very high baseline TG levels, such as familial chylomicronemia, may be left with significantly elevated on-treatment TG levels and, therefore, increased risk of acute pancreatitis despite maximal medical intervention with statins, fibrates, and omega-3 FAs. In this context, novel agents specifically targeting high TG levels are warranted to both prevent attacks of acute pancreatitis and attenuate the risk of CVD.

The rapidly evolving CV pharmacology has provided novel TG-lowering agents. Few of them have been currently approved for the management of very high TG levels, e.g., volanesorsen for the management of FCS. Most of these agents and their associated studies have alreadybeen discussed in the recent literature, including by ourselves [14,15,16].

**Figure 1:**
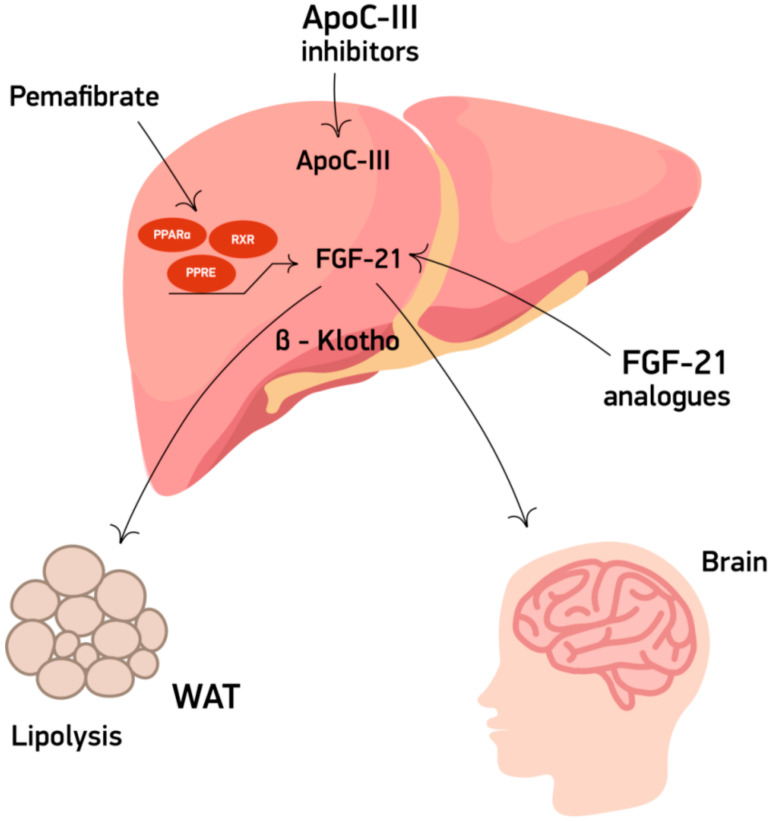
Mechanism of action of triglyceride-lowering agents ApoC-III: apolipoprotein C-III, FGF21: fibroblast growth factor 21, WAT: white adipose tissue.

In contrast, little is known about the agents which are currently in their earlier phases of development and have not been approved for use in clinical practice. In this review, we focus on available data from ongoing early-phase clinical development studies (Table 1) primarily aiming at lowering TG levels (Figure 1).

## 2. Apolipoprotein (APO) C-III Inhibitors

ApoC-III is a glycoprotein that is mainly produced by hepatocytes and to some extent by intestinal cells [17]. It binds to the surface of several lipoproteins, specifically high- density lipoprotein (HDL), low-density lipoprotein (LDL), chylomicrons, and very-low-density lipoproteins (VLDL). ApoC-IIIreduces the activity of LPL, hence inhibiting the LPL-mediated degradation of triglyceride-rich lipoproteins (TRL), as well as their hepatic endocytosis [18].Therefore, increased apoC-III activity results in elevated circulating chylomicrons and VLDL particles. Interestingly, apoC-III appears to have proatherogenic properties, as it augments the recruitment of monocytes to the vascular wall, induces an inflammatory response within the endothelial cells, and promotes LDL binding in the arterial wall [19,20].

ApoC-III LPL has been extensively studied in the context of mendelian randomization (MR) and genetic association studies to evaluate a potential causal association between elevated TG and CVD risk. The carriers of variants of the *LPL* gene with enhanced LPL activity and thus low circulating TG appear to be protected from CVD. Loss-of-function (LOF) mutations in the *APOCIII* gene also appear to confer CVD protection. A genetic analysis of 75,725 individuals who participated in two general-population studies assessed the impact of LOF mutations of the *APOIII* gene on TG levels and the subsequent CVD risk [21]. Compared with noncarriers, heterozygotes for LOF in *APOCIII* had a mean reduction in nonfasting TG levels of 44% (*p* < 0.001) [21]. The incidences of ischemic vascular disease (defined as either ischemic heart disease or ischemic cerebrovascular disease) and ischemic heart disease were reduced in heterozygotes (*p* = 0.009 and *p* = 0.05, respectively), with corresponding risk reductions by 41% (hazard ratio (HR), 0.59; 95% confidence interval (CI), 0.41 to 0.86; *p* = 0.007) and 36% (HR, 0.64; 95% CI, 0.41 to 0.99; *p* = 0.04) [21]. These observations raised the interest for the development of pharmaceutical agents aiming at significant lowering of TG levels.

The first drug specifically targeting apoC-III was volanesorsen, a chimeric antisense oligonucleotide (ASO) which inhibits the production of the *APOC-III* messenger RNA (mRNA) [22]. Volanesorsen has been evaluated in phase two and three trials and has demonstrated significant reductions in TG concentration (up to 80%), mainly in individuals with familial chylomicronemia syndrome (FCS) [23].

Ongoing trials are currently evaluating novel apoC-III-targeting therapies. ARO-APOC3 is a hepatocyte-targeted small interference RNA (siRNA) which induces the degradation of *APOC-III* mRNA. ARO-APOC3 has already been studied in a phase one trial with 12 healthy individuals. Specifically, ARO-APOC3 was administrated subcutaneously (sc) on days 1 and 29 at doses of 10, 25, and 50 mg (N = 4 in each group). All participants received two single doses. Significant reductions in APOC-III levels of 73% in the 10 mg, 90% in the 25 mg, and 94% in the 50 mg group were observed during follow-up. These changes were accompanied by significant dose-dependent reductions in TG levels by 41%, 47%, and 72% in the 10 mg, 25 mg, and 50 mg group, respectively. Furthermore, HDL cholesterol (HDL-C) levels increased with maximum elevations being reported at week 8 (+42%, +48%, +84% in the 10 mg, 25 mg, and 50 mg groups, respectively). No significant adverse events were reported apart from mild injection-site reactions and headaches [24].

The safety, tolerability, pharmacokinetics, and pharmacodynamics of single and multiple doses of scARO-APOC3 are now being evaluated in a phase one trial of healthy individuals, patients with hypertriglyceridemia (TG > 300 mg/dL; 3.38 mmol/L), and patients with a diagnosis of FCS (n = 112 participants). The number of participants with adverse events related to the drug up to day 113 is the primary endpoint of the study. Its secondary endpoints include the reductions in apoC-III levels up to day 113 and the characterization of the compound’s pharmacokinetics (time to maximum plasma concentration and terminal elimination half-life for up to 48 h after the injection) [25].

Furthermore, ARO-APOC3 administrated in twosc doses is currently being evaluated in a double-blind, placebo-controlled, phase two b study in 300 participants with severe hypertriglyceridemia, i.e., TG > 500 mg/dL (5.6 mmol/L) (based on medical history or at screening) [26]. The primary endpoint is the percent change in fasting TG at week 24, while secondary outcomes include percent change in TG levels at week 48 as well as in apoC-III, HDL-C, non-HDL-C, LDL-C, and apoB levels at week 48. Furthermore, safety will be evaluated by means of the number of participants exhibiting adverse events related to ARO-APOC3 administration [26].

Another ongoing phase one double-blind, randomized, placebo-controlled trial is currently evaluating the safety, tolerability, pharmacokinetics, and pharmacodynamics of STT-5058, a human monoclonal antibody directed against apoC-III [27]. The trial includes 104 participants, either otherwise healthy individuals with TG levels > 150 mg/dL (1.7 mmol/L) or patients with moderate hypertriglyceridemia (TG > 200 mg/dL/2.2 mmol/L). It consists of four parts: in part A, up to six single ascending intravenous (iv) doses of STT-5058 or placebo will be given in cohorts of otherwise healthy volunteers with TG > 150mg/dL; in part B, up to four multiple ascending intravenous doses of STT-5058 will be administered in otherwise different cohorts of healthy volunteers with TG > 150 mg/dL (1.7 mmol/L). These individuals will receive three of the same doses of STT-5058 or placebo at two-week intervals. Part C will include a single cohort of patients with TG > 200 mg/dL (2.2 mmol/L) who will receive three doses of STT-5058 or placebo at two-week intervals. In part D, up to two single ascending doses of subcutaneous STT-5058 will be evaluated. The primary outcome of the study is the safety and tolerability of STT-5058 at week 10 from administration [27].

This category of drugs, which aims at apoC-III reduction, seems promising, as findings from volanesorsen are encouraging [28], while a benefit in terms of atherogenesis may be anticipated as indicated by pathophysiology. Of course, the safety and efficacy in terms of CV outcomes need to be addressed and demonstrated by larger studies.

## 3. Omega-3 Fatty Acids (FA)

Omega-3 polyunsaturated FAs include the very long-chain eicosapentaenoic (EPA) and docosahexaenoic acids (DHA) as well as the shorter-chain alpha linolenic acid (ALA). Mammals do not synthesize omega-3 FAs and rely on dietary sources. The Mediterranean diet and marine foods are rich in EPA/DHA/ALA [29]. Currently, several omega-3 FAs are used in clinical practice for the treatment of severe hypertriglyceridemia, which consist of different amounts of EPA and DHA [30]. Two of these are mixtures of EPA and DHA, one contains pure EPA (icosapent ethyl), and the fourth consists of pure omega-3 carboxylic acids [30].

The role of omega-3 FAs in terms of reducing CVD risk remains controversial. There have been several randomized clinical trials (RCTs) and meta-analyses where the administration of omega-3FAs was not associated with statistically significant reductions in CVD risk [13,31,32,33]. However, a more recent positive-outcomes study with omega-3 FAs triggered again the interest for these agents. In fact, in the REDUCE-IT (cardiovascular risk reduction with icosapent ethyl for hypertriglyceridemia) study, icosapent ethyl administration was associated with a significantly reduced risk for CVD by 22% compared with the placebo in individuals with established CVD or with DM and other CV risk factors [34]. Of note, these beneficial findings occurred in the context of mild to moderate reductions in lipid parameters (TG by 19.6% and LDL-C by 6.6% vs. placebo). Therefore, it is likely that alternative mechanisms account for these favorable outcomes, including a reduction in oxidized LDL as well as in high-sensitivity C-reactive protein (hsCRP) and lipoprotein-associated phospholipase A2 (LpPLA_2_) levels [35,36].

In this context, there are several ongoing trials assessing the effects of novel omega-3 FAs. A phase one trial is currently evaluating the safety, tolerability, and pharmacokinetics of NST-1024 in 96 healthy individuals and otherwise healthy subjects with elevated TG levels (upper cutoff of TG > 150 mg/dL; 1.7 mmol/L). NST-1024 is a novel, orally administrated and chemically modified FA, manufactured from EPA. In this trial, single and multiple doses of oral NST-1024 or placebo will be administered once daily for up to 14 days. The primary outcome of the study is any reported adverse event within 4 weeks [37].

In another randomized, double-blind, placebo-controlled, phase three study, the safety and efficacy of MAT900, an investigational omega-3 FA predominantlycontaining EPA and docosapentaenoic acid 1, will be evaluated in 300 individuals with fasting triglyceridelevels ≥ 500 mg/dL (5.6 mmol/L) and <2000 mg/dL (22.5 mmol/L). Stable lipid-lowering therapy including proprotein convertase subtilisin/kexin type 9 (PCSK9) inhibitors is permitted. Capsules of MAT9001 (1 g four times daily) will be compared with placebo in terms of TG reduction within 12 weeks [38].

## 4. Fibroblast Growth Factor 21 (FGF21) Analogues

Fibroblast growth factor 21 (FGF21) is a protein, mainly synthesized in the liver, that regulates energy homeostasis and metabolism through endocrine and paracrine pathways in various tissues and organs [39]. FGF21 needs a coreceptor, β-Klotho, in order to bind on cell membrane surfaces [40]. Accordingly, the β-Klotho-FGF21 complex interacts with several receptors (FGFR1c, FGFR2c, and FGFR3c), activating intracellular tyrosine kinase pathways [41]. FGF21 signaling is mainly mediated by FGFR1c in adipose tissue, FGFR2c in the liver, and FGFR1c/3c in the pancreas [42].

FGF21 signaling has been associated with increased insulin sensitivity in adipose tissue and muscles as well as increased glucose uptake and reduced oxidative stress [39]. Through the interaction with FGFR2c, FGF21 has demonstrated beneficial effects on liver function in terms of reduced lipotoxicity and oxidative stress, reduced VLDL secretion, and increased FA oxidation [43]. Furthermore, FGF21 seems to possess anti-inflammatory properties. In animal models with nonalcoholic steatohepatitis (NASH), the administration of FGF21 analogues was associated with reduced expression of several proinflammatory cytokines, including tumor necrosis factor a (TNF-a), interleukin-6 (IL-6), IL-1, and interferon-γ (IFN-γ) [44]. Treatment with a FGF21 analogue has also been found to attenuate liver infiltration with neutrophils and macrophages in an animal model [45].

BIO89-100, a glycoPEGylated FGF21 analogue, has been evaluated in preclinical and phase one trials. The effect of BIO89-100 1 mg/kg vs. placebo on lipid parameters was evaluated in two preclinical studies in obese, diabetic monkeys. In the first study (n = 12), BIO89-100 was administrated weekly to the vehicle for 8 weeks, while in the second study (n = 12) BIO89-100 was given weekly or every other week to the vehicle for 4 weeks. Blood pressure, weight, hemoglobin A1c (HbA1c), lipid parameters, and alanine aminotransferase (ALT) were evaluated before, during, and after the administration of BIO89-100. BIO89-100 was associated with significantly reduced TG levels (by 78% in study one and by 76% in study two). Beneficial effects were also reported in several other metabolic clinical and laboratory parameters. Specifically, in the first study, BIO89-100 administration was associated with decreased LDL-C (by 37.1%), plasma glucose (by 51.4%), and body weight (by 9.3%), along with increased HDL-C levels (by 47.2%) [46].

Based on the abovementioned beneficial effects, FGF21 analogues are being evaluated as potential therapeutic agents for NASH and hypertriglyceridemia. In a phase two, randomized, double-blind, placebo-controlled trial, the efficacy and safety of BIO89-100 administrated sc weekly or every two weeks in 90 individuals with severe hypertriglyceridemia (TG > 500/1.7 mmol/L and <2000 mg/dL/22.5 mmol/L) are currently being evaluated. The primary outcome of the study is the percentage change in TG levels at week 8, while the secondary outcomesinclude percentage alterations in VLDL-C, LDL-C, non-HDL-C, HDL-C, apoB-100, remnant lipoprotein cholesterol, high-sensitivity C-reactive protein, and fasting adiponectin [30]. Overall, FGF21 analogues appear to have beneficial effects in metabolic derangements, including lipoprotein metabolism. These agents are promising therapeutic options for NASH, a common metabolic disorder for which there is an unmet clinical need, as well as for hypertriglyceridemia.

## 5. Pemafibrate

Pemafibrate is a second-generation fibrate that acts as a selective peroxisome proliferator-activated receptor alpha modulator (SPPARM-α) [47]. Through structural modifications, pemafibrate is a more potent activator of PPARαcompared with fenofibrate and possesses anti-inflammatory properties [47]. The potential role of pemafibrate in reducing residual CVD risk in individuals with dyslipidemia and type two diabetes mellitus (T2DM) is now being evaluated in the PROMINENT study (Pemafibrate to Reduce cardiovascular OutcoMes by reducing triglycerides IN diabetic patiENTs) [48]. The PROMINENT trial is now recruiting 10,000 patients with T2DM and atherogenic dyslipidaemia (mild-to-moderate hypertriglyceridemia with TG: 200–499 mg/dL; 2.26–5.64 mmol/L and low HDL-C: ≤40 mg/dL; 1.03 mmol/L). Study participants are being randomized to either pemafibrate 0.2 mg twice daily or placebo, with an average follow-up of 3.75 years. The primary endpoint is the composite of nonfatal myocardial infarction, nonfatal ischemic stroke, hospitalization for unstable angina requiring urgent coronary revascularization, and cardiovascular death, while secondary endpoints include all-cause mortality, hospitalization for heart failure, new or worsening peripheral artery disease, new or worsening diabetic retinopathy and nephropathy, and change in biomarkers including select lipid and nonlipid biomarkers and inflammatory and glycemic parameters [48].

## 6. Conclusions

Elevated circulating TGs increase the risk of CVD even in statin-treated patients, while individuals with excessive hypertriglyceridemia may suffer from severe acute pancreatitis. The development of novel TG-lowering drugs is indeed important in order to deal with residual CVD as well as acute pancreatitis. Several novel agents are now being evaluated, including apoC-III inhibitors, omega-3 FAs, and the more experimental FGF 21 agonists. However, the safety and efficacy of the abovementioned drugs should be assessed in large randomized clinical trials.

## Figures and Tables

**Table 1:** Summary of the ongoing early-phase clinical triglyceride-lowering trials.

Agent	Trial Design	Primary Endpoint	Secondary Endpoint	Inclusion Criteria	Number of Participants
ARO-APOC3 (siRNA against apoC-III)	Double-blind, placebo-controlled, phase IIb study	Percent change in fasting TG at week 24	Percent change in TG levels at week 48 as well as percent changes in apoC-III, HDL-C, non-HDL-C, LDL-C, and apoB at week 48, safety evaluation	Hypertriglyceridemia, i.e., TG > 500 mg/dL	300
ARO-APOC3 (siRNA against apoC-III)	Phase I trial of healthy individuals evaluating the safety, tolerability, pharmacokinetics, and pharmacodynamics of single and multiple doses of ARO-APOC3given s.c	Number of participants with adverse events related to the drug up to day 113	Reduction in apoC-III levels up to day 113 and several pharmacokinetic parameters (time to maximum plasma concentration and terminal elimination half time up to 48 h after the injection)	Patients with hypertriglyceridemia (TG > 300 mg/dL) and patients with a diagnosis of FCS	112
STT-5058 (monoclonal antibody against apoC-III)	Phase I double-blind, randomized, placebo-controlled trial. Consists of 4 parts. Part 1: 6 ascending i.v doses in healthy individuals with TG > 150 mg/dL. Part 2: 4 ascending i.v doses in individuals with TG > 150 mg/dL. Part 3: a single cohort of subjects with TG > 200 mg/dL who will receive 3 of the same doses at 2 week intervals. Part 4: 2 single s.c doses	Safety and tolerability of STT-5058 at week 10 from administration		Good health, BMI between 18 and 35 kg/m^2^, fasting TG between 150 and 400 mg/dL for part 3, LDL-C between 70 and 160 mg/dL	104
NST-1024 (omega-3 FA)	Phase I trial evaluating the safety, tolerability, and pharmacokinetics	Any reported adverse event within 4 weeks		Healthy individuals with elevated TG (>150 mg/dL)	96
MAT9001 (omega-3 FA)	Randomized, double blind, placebo-controlled, phase III trial	Safety and efficacy of MAT9001 in lowering TG levels in individuals with severe hypertriglyceridemia		Individuals with fasting TG ≥ 500 mg/dL and <2000 mg/dL while following therapeutic lifestyle changes and a BMI > 20 kg/m^2^	300
BIO89-100 (FGF21 analogue) (44)	Phase II, randomized, double-blind, placebo-controlled trial	Percentage change in TG levels at week 8	Alterations in VLDL-C, LDL-C, non-HDL-C, HDL-C, apoB-100, remnant lipoprotein cholesterol, high-sensitivity C-reactive protein, fasting adiponectin	Individuals with severe hypertriglyceridemia (TG > 500 and <2000 mg/dL)	90

apoB: apolipoprotein B, apoC-III: apolipoprotein C-III, BMI: body mass index, FA: fatty acids, FGF21: fibroblast growth factor 21, FCS:familial chylomicronemia syndrome, HDL-C: high-density lipoprotein cholesterol, i.v: intravenous, LDL-C: low-density lipoprotein cholesterol, VLDL: very low-density lipoprotein cholesterol, siRNA: small interference ribonucleic acid, TG: triglycerides.

## Data Availability

Not applicable.
